# The Functional Hearing Gain with an Active Transcutaneous Bone Conduction Implant Does Not Correlate with the Subjective Hearing Performance

**DOI:** 10.3390/jpm12071064

**Published:** 2022-06-29

**Authors:** Alice B. Auinger, Rudolfs Liepins, Faris F. Brkic, Erich Vyskocil, Christoph Arnoldner

**Affiliations:** Department of Otorhinolaryngology, Medical University of Vienna, Waehringer Guertel 18-20, 1090 Vienna, Austria; alice.auinger@meduniwien.ac.at (A.B.A.); rudolfs.liepins@meduniwien.ac.at (R.L.); faris.brkic@meduniwien.ac.at (F.F.B.); christoph.arnoldner@meduniwien.ac.at (C.A.)

**Keywords:** Bonebridge, bone conduction, hearing quality, quality of life, functional hearing

## Abstract

The functional hearing outcome with hearing implants does not always properly reflect the subjective benefit in everyday listening situations. In this study, the functional hearing gain and the impact on the subjective hearing ability and quality of life were assessed in patients with a Bonebridge. A chart review was performed on 45 patients with a Bonebridge who were provided with questionnaires regarding the hearing quality and health-related quality of life during their last clinical visit. The questionnaires consisted of the Speech, Spatial and Qualities (SSQ) and the Health Utility Index Mark 3 (HUI3). Eleven patients had to be excluded due to missing data. A total of 34 patients (37 ears) were included in the study. Aided hearing thresholds were significantly lower compared with the unaided condition, with a mean functional gain of 26.87 dB for patients with mixed/conductive hearing loss (MHL/CHL). Although patients with single-sided deafness (SSD) scored slightly lower on the SSQ compared with patients with MHL/CHL, all included patients reported improved subjective hearing quality with the BB compared with the hearing situation before implantation. No correlation was found between the functional hearing gain and the subdomains of the SSQ. SSD patients scored the HUI3 subdomain “hearing” slightly lower compared with MHL/CHL patients. Although not significant, a relationship was found between the functional gain and the “hearing” subdomain. No correlation was found for the other subdomains of the HUI3. Audiological measurements showed significantly improved hearing thresholds with the Bonebridge. Most importantly, the subjective benefit achieved in everyday listening situations was superior compared with the previous hearing condition. The lack of correlation between subjective questionnaire results and the functional hearing gain shows the importance of assessing both audiological and subjective hearing quality parameters in clinical routine.

## 1. Introduction

Bone conduction implants (BCIs) have been used for the treatment of conductive and mixed hearing loss (CHL and MHL, respectively) as well as single-sided deafness (SSD). Though most people benefit from conventional hearing aids, some cannot wear them due to medical reasons such as chronic otitis media or atresia of the external ear canal. One of the most frequently used implantable devices is the Bonebridge (BB), which is a semi-implantable, transcutaneous bone conduction hearing aid introduced in 2012 (MED-EL GmbH, Innsbruck, Austria). It is approved for CHL, MHL, and SSD in patients above the age of five years [1,2]. The technical design of this device circumvents the typical postoperative complications found with percutaneous implants [3,4]. Soundwaves are collected by an externally worn audio processor, which is kept in place over the implant by magnetic force. It further sends the signal to the implanted part of the device (the bone conduction floating mass transducer), where the signal is converted to mechanical vibration and delivered to the inner ear.

Although the improved hearing outcome assessed by audiological measures was previously reported [2,5], it does not reflect the patients’ subjective hearing result in everyday listening situations or their quality of life. In patients wearing a conventional hearing aid, reduced speech clarity, difficulties in noisy environments, and poor sound quality contribute to dissatisfaction and nonusage, although audiological measurements document a benefit [6]. In addition, even mild-to-moderate hearing loss is associated with emotional, social, and cognitive dysfunction, resulting in reduced quality of life (QoL) [7]. Health status measures are important as they provide an answer to the need for more sensitive outcome measures to evaluate treatment outcomes in contrast to traditional clinical outcomes, such as audiological benefit. QoL parameters have become a focus in the field of hearing amplification with hearing implants not only for the patients’ well-being, but also from an economic point of view. There is a wide range of questionnaires used for patients treated with hearing devices. The subjective benefit with either transcutaneous or percutaneous BCI has been reported by means of the Glasgow Benefit Inventory, one of the most frequently used hearing-related QoL questionnaires [8,9,10]. Data indicated that patients are more satisfied with their implant compared with their previously worn conventional hearing aid [9]. The subjective benefit with bone conduction devices was also reported by means of the Abbreviated Profile Of Hearing Aid Benefit, another frequently used hearing-related QoL questionnaire [11,12]. In a recent multicenter study reporting on long-term outcomes, patients were very satisfied or satisfied with their implant according to the hearing device satisfaction scale [13].

To date, there are limited data on functional outcomes and the impact on the quality of life of transcutaneous bone conduction devices. An instrument for assessing the health-related QoL is the Health Utility Index (HUI), which is widely used to assess the cost-effectiveness of a treatment. The HUI was recommended for the assessment of health-related QoL in patients with SSD [14], and was used to document improved QoL after cochlear implant (CI) implantation [15]. To the best of our knowledge, only one center assessed health utilities based on HUI in a small group of BCI patients, and no correlation with objective hearing measurements exists [16].

The aim of the current study was to evaluate the functional hearing gain and the subjective benefit by means of questionnaires. The HUI was used to evaluate the QoL, and a modified version of the Speech, Spatial and Qualities Questionnaire (SSQ) was used to assess the subjective hearing performance in everyday listening situations.

## 2. Material and Methods

### 2.1. Subjects

**Figure 1:**
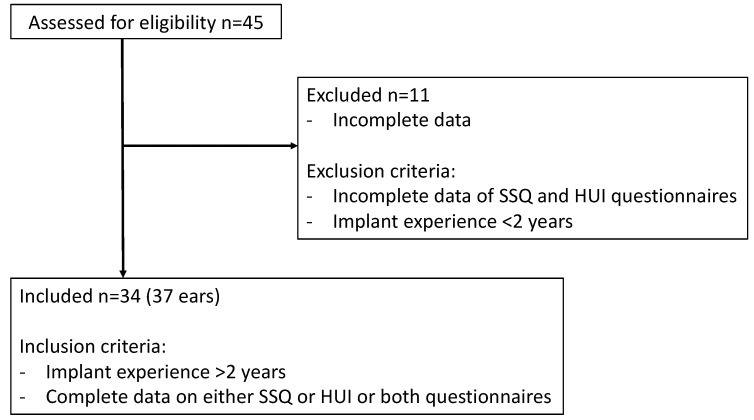
Inclusion and exclusion criteria.

A retrospective chart review was performed in patients with an active bone conduction device, a Bonebridge implant (BB, MED-EL GmbH, Innsbruck, Austria), to assess whether there was a correlation between the functional hearing gain and the subjective hearing quality and QoL. Therefore, all patients implanted with a BB at the department of Otorhinolaryngology, Head and Neck Surgery, at a tertiary care hospital between 2012 and 2016 were assessed for eligibility. Inclusion criteria were implant experience of at least two years and complete data of either the Health Utility Index Mark 3 (HUI3) or the Speech, Spatial and Qualities (SSQ) questionnaire, or both. Exclusion criteria were implant experience of less than two years and missing data of both the HUI3 and SSQ questionnaire. We excluded 11 out of 45 patients from the study due to incomplete data on both questionnaires. Subsequently, a total of 34 patients (37 ears) were included for data analysis (see Figure 1). All included patients had experience with their implant for an average of 6 years (range 3–8 years).

The following parameters were collected from patients’ charts: preoperative pure-tone thresholds for air and bone conduction, the hearing threshold in sound field in the unaided and aided conditions, word recognition scores in the unaided and aided conditions, the SSQ questionnaire, and the HUI3. Audiological data were collected from measurements performed by the institution’s audiologists. The hearing quality and QoL questionnaires were introduced in clinical routine and were answered during regular follow-up visits.

Table 1 summarizes the demographic details of the included patients. The study was approved by the institutional review board (EK 1396/2019) and conducted according to the guidelines of the Declaration of Helsinki on biomedical research involving human subjects. Informed consent was obtained from all subjects involved in the study. The examinations described in the study were part of the clinical routine, and no additional tests were performed.

### 2.2. Audiological Testing

In clinical routine, audiological data were preoperatively and postoperatively assessed during regular follow-up visits. Air-conduction (AC) thresholds were measured for octave frequencies between 125 and 6000 Hertz (Hz), and bone-conduction (BC) thresholds were measured for octave frequencies between 250 and 6000 Hz. Sound field thresholds were assessed for octave frequencies between 250 and 6000 Hz in the unaided and aided conditions. Word recognition scores (WRS) were assessed in quiet using the German Freiburger monosyllable test at 65 decibel (dB) sound pressure level (SPL) and 80 dB, both in the unaided and aided conditions.

**Figure 2:**
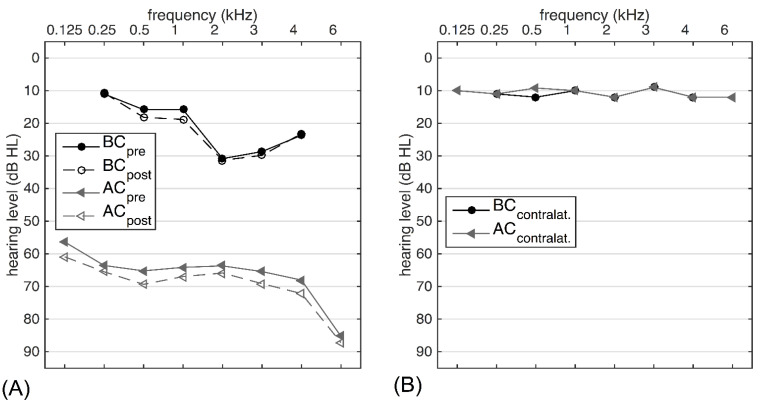
Average pure tone air-conduction (AC) and bone-conduction (BC) thresholds of the included patients. (**A**) Pre- and postoperative thresholds for patients with mixed or conductive hearing loss (N = 32) and (**B**) the contralateral preoperative thresholds of SSD patients (N = 5).

For the current study, audiological data were collected from measurements preoperatively performed and at the last clinical visit. Figure 2A shows the pre- and postoperative AC/BC thresholds for patients with CHL/MHL. In SSD patients, one of the indication criteria for the BB is a contralateral BC threshold of 20 dB hearing level (HL) or better. Figure 2B depicts the average preoperative AC and BC thresholds of the contralateral ear; none of the SSD patients had a BC threshold lower than 20 dB HL. In CHL and MHL patients, the functional gain (FG) was calculated as the difference between the aided and unaided sound field thresholds. In SSD patients, no unaided sound field thresholds were postoperatively measured during clinical routine. Therefore, the functional hearing gain was defined as the difference between the aided sound field threshold and the AC threshold at the implanted side. The WRS at 80 dB SPL in the unaided and aided conditions was not available for all included patients; therefore, only WRS at 65 dB SPL (N = 29) is reported. In all test situations, the nontest ear was masked if any functional hearing was left on the contralateral side.

### 2.3. Questionnaires

#### 2.3.1. Health Utility Index Mark 3

The HUI is a standardized questionnaire used to measure the health status and health-related QoL [17,18]. Patients recall their health status within four weeks prior to assessment. The HUI is used to describe the experience of patients undergoing therapy or long-term outcomes associated with disease or therapy, the efficacy, effectiveness, and efficiency of healthcare interventions, and the health status. It is further used to calculate quality-adjusted life years and the cost-effectiveness of treatments. Currently, two systems exist: the HUI Mark 2 (HUI2) and HUI Mark 3 (HUI3). Both include domains such as perception (vision, hearing and speech function), emotion, pain, mobility, dexterity, cognition, and self-care. The two systems are independent but complementary. Scores range from 0.0 to 1.0 indicating a health status from dead (= 0.0) to perfect (1.0). The HUI Mark 3 (HUI3), including the hearing relevant domains, was presented to patients at their last clinical visit.

#### 2.3.2. Speech, Spatial and Qualities (SSQ)

The SSQ used in this study was a modified version of the original SSQ [19], which assesses the subjective sense of listening ability and experience in everyday listening situations. It was presented to patients at their last clinical visit. Three domains are included in the questionnaire: “speech hearing” (14 questions), “spatial hearing” (17 questions), and “qualities” (18 questions). Every domain asks about the benefit of the aided condition compared with the unaided or previous aided condition with another device. For each question, a mark is placed on a scale ranging from −5 (much worse) to +5 (much better). If patients experience no difference, a mark should be put on the point 0. For questions not relevant to the patient, the answer “not applicable” should be picked.

### 2.4. Statistical Analysis

Descriptive statistics such as mean and standard deviation are used to present and summarize the data. The functional hearing gain in MHL/CHL subjects was analyzed using a paired samples t test. The subscores of the modified SSQ questionnaire were analyzed with one-sample t-tests. Prior to that, the Shapiro–Wilk test was used to confirm whether the data were normally distributed. Due to the exploratory nature of this chart review, no correction for multiplicity was applied. A *p* value < 0.05 was considered to indicate statistical significance. To examine the relationship between functional hearing gain and subjective benefit, the Spearman’s rho correlation coefficient was calculated.

## 3. Results

### 3.1. Audiological Results

**Figure 3:**
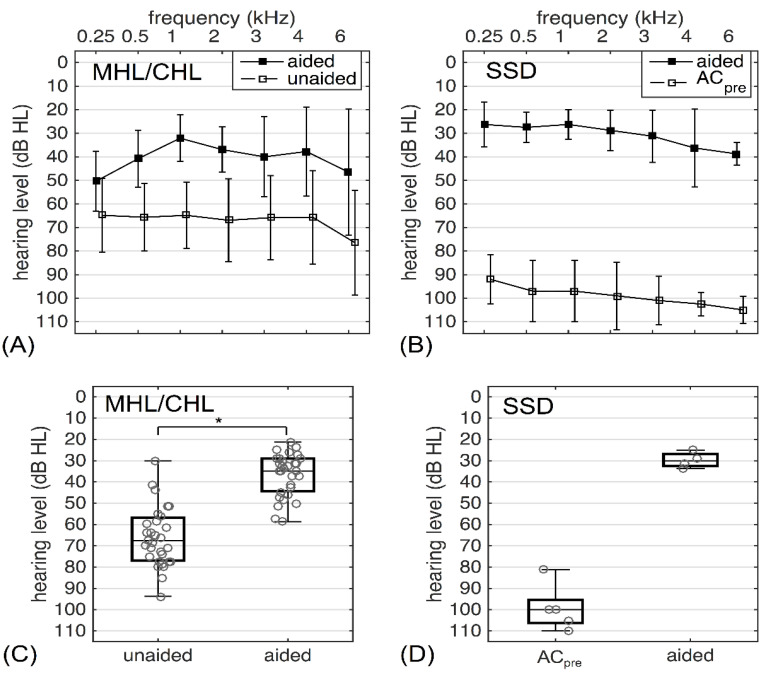
Hearing performance with and without the implant. (**A**) Average aided and unaided sound field thresholds for patients with CHL/MHL; (**B**) average aided sound field threshold and the preoperative AC threshold for SSD patients. Error bars indicate the standard deviation around the mean. (**C**,**D**) PTA_4_ defined as the average of thresholds at 0.5, 1, 2, and 4 kHz for CHL/MHL and SSD patients. Grey circles indicate individual data. * *p* < 0.0001.

Figure 3 and Table 2 depict the average unaided and aided audiological thresholds, which are separately reported for patients with MHL/CHL (*n* =32 ears) and SSD (*n* = 5 ears) for frequencies with octave steps between 0.5 and 4 kHz (PTA_4_). As only five patients with SSD could be included, no inferential statistical analysis was performed for this group due to the very small sample size. As expected, in SSD subjects, the aided thresholds were considerably better compared with the preoperative AC thresholds of the implanted side. Table 3 shows the FG with the BB. There was a statistically significant improvement in FG for patients with MHL and CHL (*p* < 0.0001).

**Figure 4:**
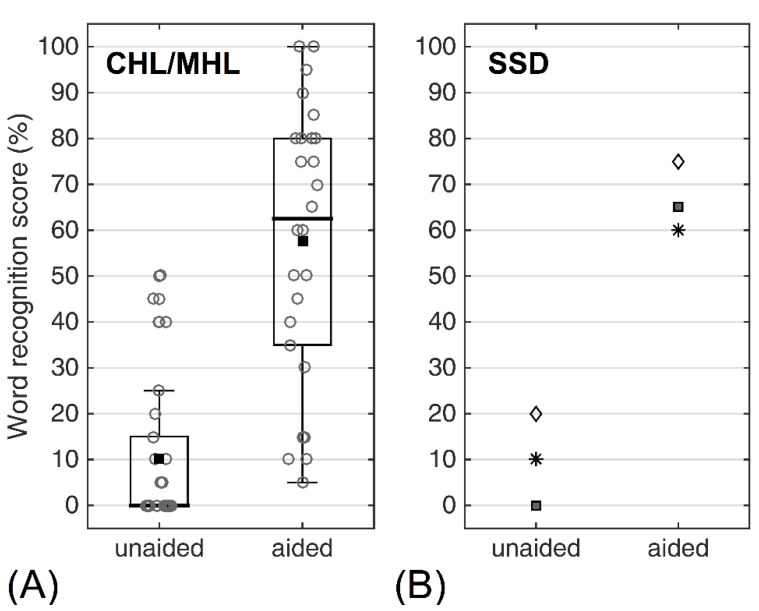
Word recognition scores with and without the BB postoperatively acquired for (**A**) patients with MHL/CHL. Bold horizontal lines indicate the median; black squares denote the mean. (**B**) SSD patients, where each symbol denotes values for a specific individual.

In total, the postoperative WRS of 29 patients was available for analysis, and are summarized in Figure 4. For the CHL/MHL patients, the mean unaided WRS at 65 dB SPL was 10.2 ± 24%, and 57.7 ± 30% in the aided condition (*n* = 26), yielding an average increase of 47.5%. WRS data were available for three SSD patients. In these patients, the mean increase at 65 dB SPL was 58% from 10% ± 7% unaided to 68% ± 11% aided.

### 3.2. Subjective Hearing Quality

#### 3.2.1. SSQ—Speech, Spatial and Qualities

**Figure 5:**
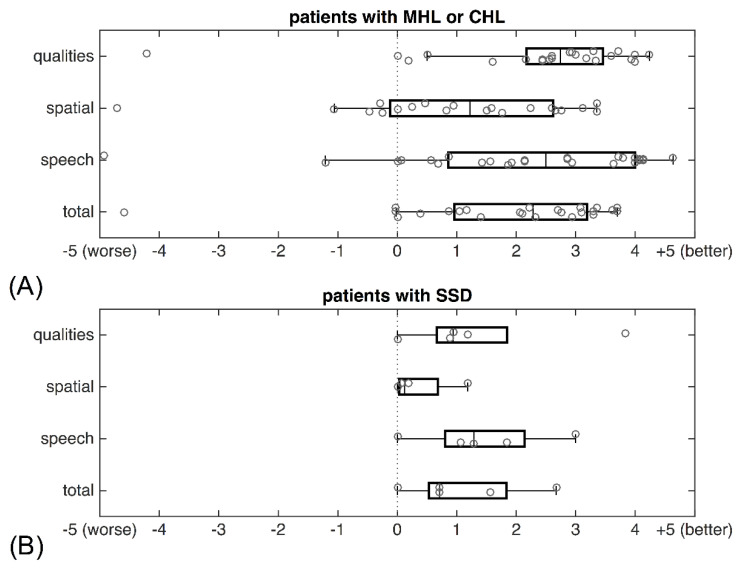
Modified SSQ questionnaire total score and subscores for (**A**) CHL/MHL patients (N = 24) and (**B**) patients suffering from SSD (N = 5). Grey circles depict individual data. The value “0” on the *x*-axis represents no difference compared with the situation prior to implantation. Positive values indicate subjective improvement; negative values indicate deterioration of the hearing situation.

The SSQ was used to assess the subjective hearing quality in everyday listening situations with the BB compared with the hearing situation before implantation. The total scores and subscores from the last clinical visit are separately depicted for CHL/MHL and SSD patients in Figure 5. If more than 10% of questions of each subdomain were not answered, the subdomain and total scores were rejected. Of all included patients, the total scores of 25 CHL/MHL patients and 5 SSD patients were available. Generally, SSD patients scored slightly lower compared with patients with CHL/MHL. The highest score was assessed in the domain “qualities” in patients with CHL/MHL. The “spatial hearing” domain was scored lowest in both the CHL/MHL and SSD patients. One-sample t-tests were used to assess whether the SSQ scores were significantly different from zero, i.e., if there was a difference in subjective hearing benefit after and before implantation. Our analysis revealed a statistically significant improvement in the total score and all three subscores (see Table 4). The results of correlation analysis revealed no distinct relationship between the FG and the speech (rc = 0.19, *p* = 0.35), spatial (rc = −0.19, *p* = 0.42), or qualities (rc = 0.11, *p* = 0.61) subdomains of the SSQ.

#### 3.2.2. Health Utility Index

HUI3 data were available from all 34 included patients who answered the questionnaire at their last clinical visit. Table 5 shows results for each domain. Again, no significant correlation was found between the subdomains of the HUI3 and the FG, but a statistical trend was found between the FG and the subdomain “hearing”. Correlation coefficients are depicted in Table 5.

## 4. Discussion

The current audiological outcome is comparable to previous reported hearing gains with bone conduction devices. For patients with CHL/MHL, mean FGs have been reported to be 19.8–50.0 dB, and improvements in WRS ranged from 29.4% to 70.0% [11,20,21,22,23]. Patients with SSD, in contrast, improved by 11.1–21.6 dB, and the speech reception increased by 4.7–40.7% [20,21]. In the current study, a total of eight patients had a WRS less than 50% at 65 dB in the aided condition, of which five did not even reach 30% at 65 dB. Nevertheless, this result can be assumed as partial success because WRS scores were 0% to 5% in the unaided condition and 35% to 70% at 80 dB with their implant. Five of them had an atresia of the outer ear canal, of which one could only achieve an aided threshold of 15% at 65 dB with a previous worn bone conduction hearing aid; another patient could achieve 10% at 65 dB with a previously worn BAHA; and a third patient was not sufficiently amplified with hearing glasses. Two of the patients with atresia did not wear a hearing aid before implantation of the BB at an age around 14 and 19 years, suggesting that delayed hearing rehabilitation influences speech performance. Three patients had cholesteatoma, with sensorineural hearing thresholds being borderline to BCI indication criteria.

Audiological data do not represent the individual hearing performance in everyday listening situations. How specific aspects of hearing are perceived by patients is frequently measured with the SSQ. Previously published data mostly reported on the original SSQ, in which patients score on a visual analogue scale between 0 and 10, representing complete inability to complete ability in different hearing situations. In the current study, the objective was to assess the subjective hearing benefit with the BB compared with the previous hearing situation, whether it was with a conventional hearing aid, a bone conduction hearing aid, or another implant. The results of the SSQ were generally positive, indicating that patients experienced subjective benefit with the BB compared with the previous hearing situation. Interestingly, the positive results on the SSQ did not correlate with the FG, indicating the importance of assessing both audiological and subjective measurements. Den Besten et al. reported improvements on all subscales, namely “speech hearing”, “spatial hearing”, and “qualities” with the Baha Attract System, though slightly lower pre- and postoperative scores and less improvement were found in SSD patients compared with listeners with CHL/MHL [20]. As in the current study, the lowest scores were found for “spatial hearing”.

The highest scores were assessed for the “speech” and “qualities” domains for patients with CHL/MHL, but slightly lower scores for patients with SSD. These findings are also in line with the results reported by Hougaard et al., with lowest scores in the “spatial” domain for both the CHL/MHL and SSD patients [21]. Laske et al. evaluated SSD patients who were implanted with a Bonebridge. After a learning curve of six months, the subjective benefit was generally rated positive, but scores in the spatial and qualities sections were close to zero [24]. Even though a clear subjective benefit with the BB can be assessed, the score on the “spatial hearing” domain is comparatively low. This can be explained by the fact that the device does not provide true binaural hearing. This subjective benefit further diminished in patients with SSD.

A systematic review found limited evidence regarding the QoL benefit of bone-anchored hearing aids [10]. The HUI3, as a measure of general health status used in the current study, was answered by the patients during their last clinical visit. As we began assessing the questionnaire within clinical routine after patients were implanted with the BB, no preoperative data were available. The impact of the actual intervention therefore cannot be properly assessed; we compared results with those in the literature. The advantage of the HUI in general is that it meets the criteria for utility scores used to calculate quality adjusted life years to evaluate cost-utilities [18]. The general utility scores of the HUI3 were 0.68 for the CHL/MHL patients wearing a BB and 0.58 for SSD patients with a BB. The scores are slightly lower compared with previously reported health states based on HUI3 [16].

Looking closer to the “hearing” domain, patients with CHL/MHL scored 0.65. This is slightly lower than for a group of patients examined by den Besten [20]. The authors included patients with BAHA Attract, and preoperatively assessed the HUI3 and six months after implantation. The score of the “hearing” domain significantly improved from 0.62 to 0.77, while the improvement in patients with SSD was not significant (0.73 preoperatively vs. 0.76 postoperatively). In the current study, SSD patients scored lower, with 0.55. The scores for the “speech” domain are comparable between the two studies (0.98 vs. 1.00 in the current study).

A study performed by Schwartz et al. showed a “higher” multi-attribute score of 0.70 based on the HUI3 for unilateral deaf patients with a BAHA compared with the current results [25]. This difference might have been caused by including patients with serviceable residual hearing in the implanted ear with slightly higher utility scores compared with patients with complete deafness (0.84 vs. 0.62). Most patients with SSD are nowadays implanted with a cochlear implant (CI). Some authors have examined the impact of a CI on the health status of SSD patients. Czerniejewska-Wolska et al. reported a multiattribute score of 0.56 one year after CI implantation [15]. Unfortunately, they did not report on the hearing status on the contralateral side. Arndt et al. reported a higher multiattribute score of 0.80 in CI patients with SSD [26]. Although there was no significant difference between the subdomains, the overall score was significantly higher compared with the unaided situation or with a previous worn CROS hearing aid or BAHA. It seems that the amount of hearing loss correlates with lower HUI3 scores. Francis et al. retrospectively assessed the HUI3 in CI patients [27]. Patients were asked to recall their health status before CI surgery and at the time of the assessment. The HUI3 multiattribute score increased by 0.24 from 0.37 preoperatively to 0.61 postoperatively. The largest increase was found for the “hearing” (0.70 to 0.85) and “emotion” (0.85 to 0.96) subdomains; speech perception scores were found to be predictive of the impact of CI on the QoL. In the current study, SDD patients with a BB scored 0.55 in the domain “hearing”. We assume that the lack of true binaural hearing might account for this difference. Another study conducted by Manrique-Huarte et al. revealed a HUI3 multiattribute score of 0.57 in patients who were provided with a CI, while a control group with profound sensorineural hearing loss achieved only 0.45 [28].

The bias of the current study resulting from recalling the hearing situation before the surgery can be probably neglected as other authors came to similar QoL results, whether it was retrospectively or prospectively assessed [29,30]. Patients with a BB regularly experience what it feels like when hearing loss reoccurs because they wear a hearing device that is turned off in specific situations [30].

In conclusion, patients with a BB significantly improved in their aided hearing condition compared with the unaided situation. Although patients with a BB rated their subjective hearing quality better compared with their previous hearing situation, no significant correlation with the FG was observed. Audiological measurements do not sufficiently reflect the subjective benefit in everyday listening situations. Therefore, hearing-related questionnaires should be provided during clinical routine. In terms of health-related QoL, a statistical trend for the “hearing” subdomain of the HUI3 and the FG could be observed.

## Figures and Tables

**Table 1:** Bonebridge users’ demographics.

Participants	*n* = 34 (37 Ears)
**Sex**	
Male	17 (50.0%)
Female	17 (50.0%)
**Age**	
Age at implantation	38 +/- 20 years
Age at follow-up	43 +/- 20 years
**Indication**	
Conductive	18 (50.0%)
Mixed	14 (40.0%)
Single-sided deafness	5 (10.0%)
**Cause**	
Atresia/anotia	20
Chronic otitis media	10
Otosclerosis	1
Squamous cell carcinoma	1
SSD	5
Meningitis (*n* = 1)	
Congenital hearing loss (*n* = 2)	
Sudden hearing loss (*n* = 2)	
**Previous used hearing device**	
None	17
BAHA headband	3
Hearing aid	5
Bone conduction hearing aid	2
Stapes prothesis	1
Vibrant Soundbridge	2
CROS hearing aid	1
Xomed Audiant	1
Sophono	2
BAHA implant	1
Bone conduction spectacles	2

**Table 2:** Audiologic results.

	Mixed/Conductive (*n* = 32)		SSD (*n* = 5)	
	PTA_preop_	PTA_postop_	PTA_preop_	
AC	66 ±19	68 ± 15	99 ± 10	
BC	21 ± 15	23 ±13		
	PTA_unaided_	PTA_aided_		PTA_aided_
	66 ± 14	37 ± 10		30 ± 4

The pure tone average was preoperatively computed for frequencies 0.5, 1, 2, and 4 kHz (PTA_preop_), postoperatively at last follow-up (PTA_postop_), in the unaided (PTA_unaided_) and aided (PTA_aided_) conditions in dB HL. AC, air conduction; BC, bone conduction; SSD, single-sided deafness. SSD patients had no measurable BC thresholds.

**Table 3:** Functional gain.

	Mean Difference	Lower 95% CI	Upper 95% CI	df	*p*-Value
PTA_4_ BCI aided vs. unaided	26.9 dB	23.2	34.6	33	<0.0001

PTA_4_ BCI aided vs. unaided for MHL/CHL patients; paired sample t-test.

**Table 4:** Speech, spatial, and qualities.

	Mean Difference	Lower 95% CI	Upper 95% CI	df	*p*-Value
SSQ12 total	1.85	1.07	2.63	23	<0.0001
SSQ12 speech	2.15	1.29	3.02	25	<0.0001
SSQ12 spatial	1.03	0.13	1.93	19	0.027
SSQ12 qualities	2.38	1.61	3.15	23	<0.0001

Mean difference as distance from 0 for CHL/MHL patients, one-sample *t*-test.

**Table 5:** Health Utility Index.

HUI3	Conductive/Mixed *N* = 29	SSD *N* = 5	Rc FG

Multi	0.68 ± 0.25	0.58 ± 0.30	−0.097 *p* = 0.622
Vision	0.98 ± 0.02	0.98 ± 0.03	−0.051 *p* = 0.797
Hearing	0.65 ± 0.30	0.55 ± 0.38	−0.331 *p* = 0.085
Speech	1.00 ± 0.00	1.00 ± 0.00	
Ambulation	0.93 ±0.22	1.00 ± 0.00	0.087 *p* = 0.661
Dexterity	0.98 ± 0.06	0.98 ± 0.05	0.168 *p* = 0.393
Emotion	1.00 ± 0.00	1.00 ± 0.00	
Cognition	0.94 ± 0.07	0.97 ± 0.06	0.042 *p* = 0.833
Pain	0.90 ± 0.14	0.78 ± 0.44	−0.026 *p* = 0.897

Mean HUI3 scores ± SD for patients with CHL/MHL and SSD.

## Data Availability

Data will be made available upon reasonable request.

## References

[1] Sprinzl G., Lenarz T., Ernst A., Hagen R., Wolf-Magele A., Mojallal H., Ingo T., Robert M., Mario W.D. (2013). First European multicenter results with a new transcutaneous bone conduction hearing implant system: Short-term safety and efficacy. Otol. Neurotol..

[2] Baumgartner W.-D., Hamzavi J.-S., Böheim K., Wolf-Magele A., Schlögel M., Riechelmann H., Zorowka P., Koci V., Keck T., Potzinger P. (2016). A New Transcutaneous Bone Con-duction Hearing Implant: Short-term Safety and Efficacy in Children. Otol. Neurotol..

[3] Hobson J.C., Roper A.J., Andrew R., Rothera M.P., Hill P., Green K.M. (2009). Complications of bone-anchored hearing aid implantation. J. Laryngol. Otol..

[4] Reyes R.A., Tjellström A., Granström G. (2000). Evaluation of implant losses and skin reactions around extraoral bone-anchored implants: A 0- to 8-year follow-up. Otolaryngol Head Neck Surg..

[5] Brkic F.F., Riss D., Scheuba K., Arnoldner C., Gstöttner W., Baumgartner W.-D., Vyskocil E. (2019). Medical, Technical and Audiological Outcomes of Hearing Rehabilitation with the Bonebridge Transcutaneous Bone-Conduction Implant: A Single-Center Experience. J. Clin. Med..

[6] McCormack A., Fortnum H. (2013). Why do people fitted with hearing aids not wear them?. Int. J. Audiol..

[7] Mulrow C.D., Aguilar C., Endicott J.E., Velez R., Tuley M.R., Charlip W.S., Hill J.A. (1990). Association Between Hearing Impairment and the Quality of Life of Elderly Individuals. J. Am. Geriatr. Soc..

[8] Bianchin G., Bonali M., Russo M., Tribi L. (2015). Active Bone Conduction System: Outcomes with the Bonebridge Transcutaneous Device. ORL.

[9] Monini S., Bianchi A., Talamonti R., Atturo F., Filippi C., Barbara M. (2016). Patient satisfaction after auditory implant surgery: Ten-year experience from a single implanting unit center. Acta Oto-Laryngologica.

[10] Johnson C.E., Danhauer J.L., Reith A.C., Latiolais L.N. (2006). A Systematic Review of the Nonacoustic Benefits of Bone-Anchored Hearing Aids. Ear Hear..

[11] Skarżyński P.H., Ratuszniak A., Król B., Kozieł M., Osińska K., Cywka K.B., Sztabnicka A., Skarżyński H. (2019). The Bonebridge in Adults with Mixed and Conductive Hearing Loss: Audiological and Quality of Life Outcomes. Audiol. Neurotol..

[12] Oh S.-J., Goh E.-K., Choi S.-W., Lee S., Lee H.M., Lee I.-W., Kong S.-K. (2019). Audiologic, surgical and subjective outcomes of active transcutaneous bone conduction implant system (Bonebridge). Int. J. Audiol..

[13] Chung K. (2004). Challenges and recent developments in hearing aids. Part II. Feedback and occlusion effect reduction strategies, laser shell manufacturing processes, and other signal processing technologies. Trends Amplif..

[14] Van de Heyning P., Távora-Vieira D., Mertens G., Van Rompaey V., Rajan G.P., Müller J., Hempel J.M., Leander D., Polterauer D., Marx M. (2016). Towards a Unified Testing Framework for Single-Sided Deafness Studies: A Consensus Paper. Audiol. Neurootol..

[15] Czerniejewska-Wolska H., Kałos M., Gawłowska M., Sekula A., Mickiewicz P., Wiskirska-Woźnica B., Karlik M. (2019). Evaluation of quality of life in patients after cochlear implantation surgery in 2014–2017. Otolaryngol. Polska.

[16] Quality O.H. (2020). Implantable Devices for Single-Sided Deafness and Conductive or Mixed Hearing Loss: A Health Technology As-sessment. Ont Health Technol. Assess Ser..

[17] Furlong W.J., Feeny D.H., Torrance G.W., Barr R.D. (2001). The Health Utilities Index (HUI^®^) system for assessing health-related quality of life in clinical studies. Ann. Med..

[18] Horsman J., Furlong W., Feeny D., Torrance G. (2003). The Health Utilities Index (HUI): Concepts, measurement properties and applications. Health Qual. Life Outcomes.

[19] Gatehouse S., Noble W. (2004). The Speech, Spatial and Qualities of Hearing Scale (SSQ). Int. J. Audiol..

[20] Den Besten C.A., Monksfield P., Bosman A., Skarzynski P.H., Green K., Runge C., Wigren S., Blechert J.I., Flynn M.C., Mylanus E.A.M. (2019). Audiological and clinical outcomes of a transcutaneous bone conduction hearing implant: Six-month results from a multicentre study. Clin. Otolaryngol..

[21] Hougaard D.D., Boldsen S.K., Jensen A.M., Hansen S., Thomassen P.C. (2017). A multicenter study on objective and subjective benefits with a transcutaneous bone-anchored hearing aid device: First Nordic results. Eur. Arch. Oto-Rhino-Laryngol..

[22] Garcier M., Lavedrine A., Gagneux C., Eluecque T., Bozorg Grayeli A. (2021). Bone-Anchored and Closed Skin Bonebridge Implant in Adults: Hearing Performances and Quality of Life. Audiol. Neurootol..

[23] Sprinzl G., Lenarz T., Hagen R., Baumgartner W.D., Keintzel T., Keck T., Riechelmann H., Magele A., Salcher R., Maier H. (2021). Long-Term, Multicenter Results With the First Transcutaneous Bone Conduction Implant. Otol. Neurotol..

[24] Laske R.D., Röösli C., Pfiffner F., Veraguth D., Huber A.M. (2015). Functional Results and Subjective Benefit of a Transcutaneous Bone Conduction Device in Patients With Single-Sided Deafness. Otol. Neurotol..

[25] Schwartz S.R., Kobylk D. (2016). Outcomes of Bone Anchored Hearing Aids (BAHA) for Single Sided Deafness in Nontraditional Candidates. Otol. Neurotol..

[26] Arndt S., Aschendorff A., Laszig R., Beck R., Schild C., Kroeger S., Ihorst G., Wesarg T. (2011). Comparison of Pseudobinaural Hearing to Real Binaural Hearing Rehabilitation After Cochlear Implantation in Patients With Unilateral Deafness and Tinnitus. Otol. Neurotol..

[27] Francis H.W., Chee N., Yeagle J., Cheng A., Niparko J.K. (2002). Impact of Cochlear Implants on the Functional Health Status of Older Adults. Laryngoscope.

[28] Manrique-Huarte R., Calavia D., Irujo A.H., Girón L., Manrique-Rodríguez M. (2016). Treatment for Hearing Loss among the Elderly: Auditory Outcomes and Impact on Quality of Life. Audiol. Neurotol..

[29] Palmer C.S., Niparko J.K., Wyatt J.R., Rothman M., de Lissovoy G. (1999). A prospective study of the cost-utility of the multichannel cochlear implant. Arch. Otolaryngol. Head Neck Surg..

[30] Cheng A.K., Niparko J.K. (1999). Cost-utility of the cochlear implant in adults: A meta-analysis. Arch. Otolaryngol. Head Neck Surg..

